# Analysis of the influencing factors of the scientific fitness literacy of nurses in the context of exercise and medicine integration

**DOI:** 10.1186/s12909-024-05378-2

**Published:** 2024-04-11

**Authors:** Juan Liu, Man-Hui Hu, Xuan Bai, Yu Zhao, Cai-Hong Cui, Yan Wang, Xiao-Yang Shi, Zong-Bao Niu

**Affiliations:** 1 Department of Rehabilitation Medicine, Affiliated Hospital of Hebei University, Baoding, Hebei 071000 China; 2 Department of Gastrointestinal Surgery, Affiliated Hospital of Hebei University, Baoding, Hebei 071000 China; 3 Development Planning Office, Affiliated Hospital of Hebei University, Baoding, Hebei 071000 China; 4 Hebei University Health Science Center, Baoding, Hebei 071000 China; 5 College of Nursing, Hebei University, No.342, Yuhua East Road, LianChi District, Baoding City, Hebei Province 071000 China; 6 Department of Ultrasound Diagnosis, Affiliated Hospital of Hebei University, Baoding, Hebei 071000 China

**Keywords:** Exercises and medicine integration, Nurse, Scientific fitness literacy, Influencing factor, Socioecology model

## Abstract

**Objective:**

The present study aims to explore the influencing factors of the scientific fitness literacy of nurses and provide a strategic basis for literacy improvement.

**Methods:**

A questionnaire on the influencing factors of scientific fitness literacy of nurses was designed by the group conducting the present study; the questionnaire was based on the socioecology model and the questionnaire preparation method. The general data questionnaire and the questionnaire on the influencing factors of scientific fitness literacy of nurses were adopted to investigate nurses in tertiary hospitals in order to analyze and discuss the influencing factors of their scientific fitness literacy.

**Results:**

(1) The questionnaire on the influencing factors of the scientific fitness literacy of nurses comprised five dimensions and 36 items. The overall item-content validity index was 0.833–1.000, the scale-content validity index was 0.974, and the overall Cronbach’s α coefficient was 0.955; (2) the results of the pairwise Pearson correlation analysis showed that all five dimensions were positively correlated with the scientific fitness literacy of nurses; and (3) the results of the multiple linear regression analysis revealed that five dimensions, as well as the existence of exercise habits in daily life, had a significant impact on the scientific fitness literacy of nurses (*P* < 0.001).

**Conclusion:**

The factors influencing the scientific fitness literacy of nurses involved all levels of the socioecological system. The methods of improving the awareness of the scientific fitness of nurses and providing opportunities for scientific fitness activities via the hospital played a critical role in literacy improvement. However, the lack of professional guidance and an atmosphere promoting scientific fitness might hinder literacy improvement.

## Introduction

The “exercise is a good doctor” concept has gradually taken root throughout the population, and an increasing number of people recognize scientific exercise as one of the most economical and practical means of disease prevention and health-promoting treatments [1, 2]. However, with the acceleration of urbanization comes a failure to reach requirements in terms of exercise time, intensity, and items; furthermore, ineffective exercise, injury, and failure to persist during exercise frequently occur [3]. Exercise without a scientific base may lead to injury of joints, muscles, bones, etc., even sudden cardiac death, acute myocardial infarction, and other problems [3]. The incidence of the above adverse events has been increasing year by year [4, 5]. This indicates that the current public lack of awareness and ability of scientific fitness, which will affect the health benefits of exercise. Therefore, we need to find more effective ways to promote people to take scientific exercise.

During this challenge, medical practitioners are the most trusted source of information for patients. They can educate patients about healthy behaviors while also providing services concerning the national health-related policy [6, 7]. Nurses are the largest group of staff in the medical and health system. In the context of exercise and medicine integration, health education and guidance related to scientific fitness conducted by nurses may not only respond to national policies but also have practical feasibility [6, 7]. Nurses undertake the work of health education and health promotion, and are the caregivers of patients. Contact a large number of patients and their families in daily work, and often discuss healthy behaviors with them. In the future, nurses will play an increasingly important role in guiding patients on scientific exercise. Exploring and analyzing the influencing factors of the scientific fitness literacy (i.e., the notion of properly doing exercise) of nurses would be of great significance to the scientific fitness of nurses and patients as well as to health promotion, disease therapy, and rehabilitation.

**Fig. 1:**
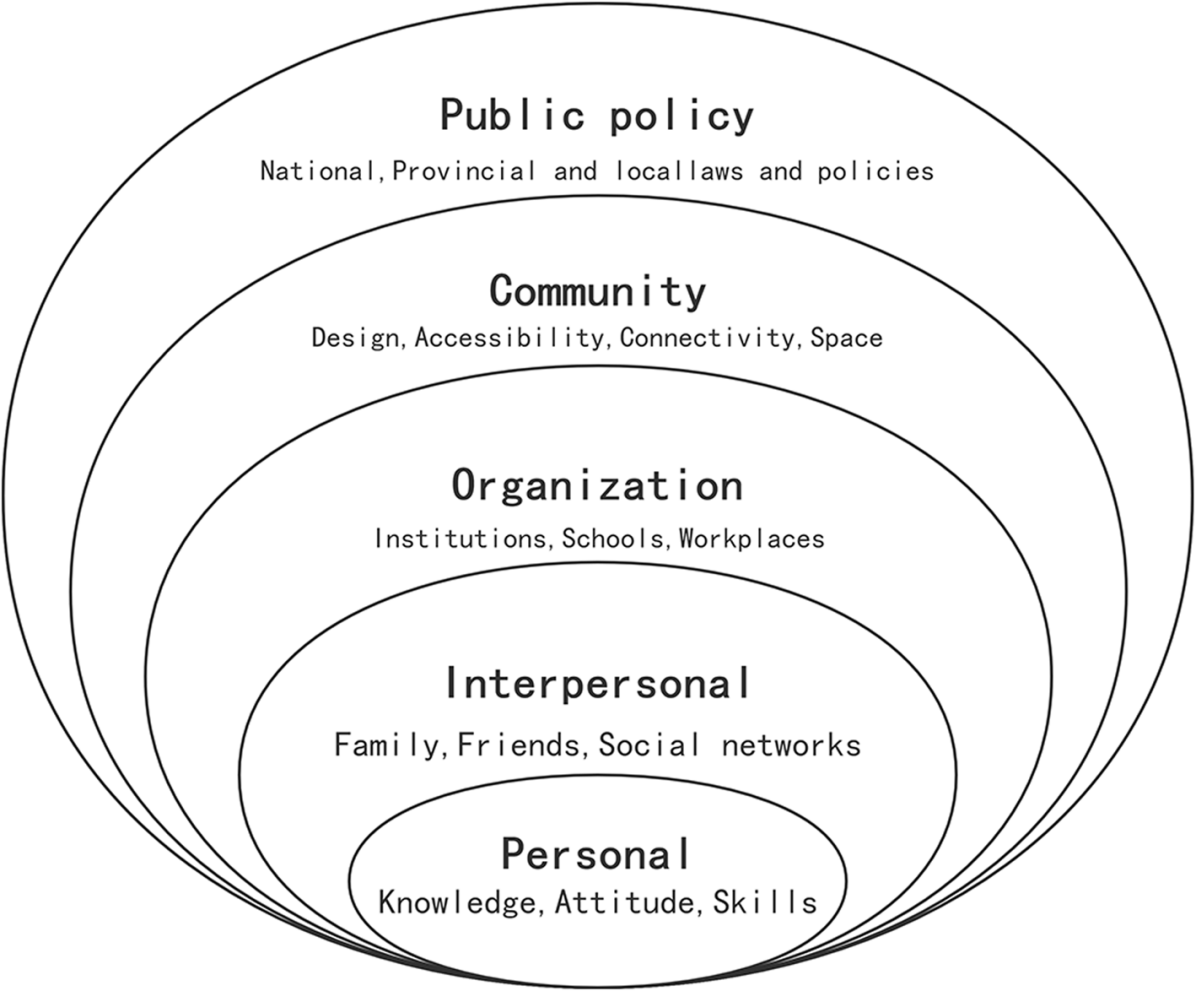
Socioecological model

The social ecosystem theory focuses on placing individuals in the environmental system, emphasizes the influence of various factors on individuals, regards the social environment of human existence as a social ecosystem, emphasizes the importance of ecological environment (human survival system) for the analysis and understanding of individual behavior, and reveals the important influence of family and social system on individual behavior. With the development and improvement of the socioecological system theory [8, 9], a relatively perfect socioecological model has been formed (Fig. 1). In this model, the microscopic, mesoscopic, and macroscopic systems are specifically divided into five dimensions: (1) individual, (2) interpersonal, (3) organizational, (4) community, and (5) policy to investigate the factors associated among people, the environment and things. Berkman [10] explained that the distal level might have a broader impact on individuals than the other dimensions; it could modify the relationship between the environment and the individual. The influencing factors of scientific fitness literacy discussed in this study involved multiple levels. The socioecological model could comprehensively summarize the influencing factors of knowledge, attitudes, skills, and behaviors concerning scientific fitness. The theory has been applied to the analysis of college students’ health literacy, the analysis of the status quo of adolescent interactive health literacy in poor areas and the literacy of scientific medical consultation [11–13]. So the objective of this study was to explore the influencing factors of the scientific fitness literacy of nurses and provide a strategic basis for literacy improvement.

## Methods

### Development of the questionnaire on the influencing factors of the scientific fitness literacy of nurses

#### Formation of the first draft of the questionnaire on the influencing factors of the scientific fitness literacy of nurses (Table 1)

**Table 1:** Structure form of the influencing factors of scientific fitness literacy of nurses

Dimension	Content
Individual	The perceptions, attitudes, behaviors, skills, enjoyment, motivation, and self-efficacy about exercise in the individuals as well as the demographic features
Interpersonal	The influence of family/friends/colleagues, social support, the influence of sports professionals, and community culture
Organizational	The implementation of the idea of sports and medicine integration by the hospital where the nurse works, and the fitness activities provided by the hospital
Community environment	The availability and convenience of community fitness facilities
Policy	The awareness of physical education policies, physical education classes, and policies related to the implementation of scientific fitness

The basic composition of the five dimensions was formed in strict accordance with the requirements of questionnaire compilation, based on the theoretical model of socioecology, and combined with theoretical analysis and related literature review. The author discussed the prepared items with the research group item by item. The members participating in the group discussion included one professor, two associate professors, one lecturer, and three graduate students. The above discussion enabled the language in the questionnaire to be more scientific and refined, enhanced operability, and increased the questionnaire’s coherency by improving the item content.

Expert consultation was then conducted. Six experts were invited to evaluate the questionnaire items. These experts specialized in exercise rehabilitation, cardiac rehabilitation, physical training, etc., had senior professional titles, and were concurrently employed in relevant positions, such as the exercises and health sub-committee of the Chinese Preventive Medicine Association and the Chinese Cardiac Rehabilitation Alliances and Rehabilitation Physician Nursing alliances. A Likert 4-point scale was adopted in the expert consultation form: 4 points = very relevant; 3 points = very relevant but not requiring revision; 2 points = need for revision, otherwise irrelevant; and 1 point = totally irrelevant. The item-content validity index (I-CVI) was adopted as the item screening criterion. The item was considered retained when the number of experts was ≥ 6 and I-CVI was ≥ 0.78. The results of the expert consultation were summarized, and after discussion among the study group, individual items were revised following expert opinions. The revised newly added five items were redistributed to six experts via email and WeChat. These six experts maintained the original attitude towards the revised items (Table 2).

**Table 2:** The analysis results of scale extreme value test and the correlation between each item and the total score(*n* = 300)

Item	The analysis results of the extreme value test		The correlation between each item and the total score		Item	The analysis results of the extreme value test		The correlation between each item and the total score	
	*t*	*P*	r	*P*		*t*	*P*	r	*P*
01	9.405	0.000	0.475	0.000	20	13.784	0.000	0.668	0.000
02	3.467	0.001	0.257	0.001	21	9.515	0.000	0.574	0.000
03	3.911	0.000	0.374	0.000	22	12.461	0.000	0.666	0.000
04	11.241	0.000	0.480	0.000	23	19.567	0.000	0.695	0.000
05	8.344	0.000	0.399	0.000	24	21.448	0.000	0.727	0.000
06	-1.922	0.056	-0.172	0.003	25	10.254	0.000	0.627	0.000
07	7.236	0.000	0.398	0.000	26	15.262	0.000	0.668	0.000
08	9.240	0.000	0.458	0.000	27	18.284	0.000	0.688	0.000
09	5.800	0.000	0.349	0.000	28	24.998	0.000	0.728	0.000
10	9.630	0.000	0.465	0.000	29	17.669	0.000	0.679	0.000
11	13.298	0.000	0.541	0.000	30	15.798	0.000	0.672	0.000
12	11.481	0.000	0.531	0.000	31	21.104	0.000	0.719	0.000
13	15.663	0.000	0.610	0.000	32	34.871	0.000	0.759	0.000
14	21.406	0.000	0.630	0.000	33	34.778	0.000	0.785	0.000
15	16.095	0.000	0.599	0.000	34	31.623	0.000	0.780	0.000
16	20.456	0.000	0.679	0.000	35	25.110	0.000	0.766	0.000
17	8.221	0.000	0.555	0.000	36	31.623	0.000	0.792	0.000
18	8.202	0.000	0.598	0.000	37	29.081	0.000	0.789	0.000
19	17.157	0.000	0.728	0.000	38	29.081	0.000	0.777	0.000

The five newly added items were considered strongly relevant by five experts and relatively strongly relevant by one expert. The I-CVI of the questionnaire on the influencing factors was 0.833–1.000, and the content validity index (CVI) of the questionnaire was 0.965. Thus, the first draft of the questionnaire comprised five dimensions, and 38 items were formed.

#### Formation of the official draft of the questionnaire on the influencing factors of the scientific fitness literacy of nurses

The questionnaire was designed and compiled based on the factors covered in the five dimensions mentioned above and with the relevant literature review and the adoption of the socioecology model to explore the influencing factors of the scientific fitness literacy of nurses.

### Study objects

The method of convenience sampling was adopted to select the nurses of the tertiary hospitals in Baoding as the investigation objects.

The inclusion criteria were as follows: nurses with a Nurse Practitioner Certificate within the valid registration period; nurses in clinical frontline nursing or nursing management; nurses who had not participated in similar studies recently; and nurses who knew the purpose of the study and volunteered to participate in the survey.

The exclusion criteria were as follows: intern nurses and nurses with advanced study; and nurses who were not in the hospital due to maternity leave or vacation during the investigation.

### Sample size

In this study, we used the empirical method combined with 5–10 times the questionnaire items to calculate the sample size. For the investigation, we selected 300 nurses who met the inclusion criteria and those who did not meet the exclusion criteria. According to the collected questionnaire data, we invited experts to conduct item analysis, and at the same time, we tested the reliability and validity. This systematic approach aims to ensure the reliability and validity of the findings.

### Survey tool

Questionnaire for general data: age, gender, educational background, place of residence, marital status, body mass index (BMI) (kg/m^2^), length of service, job title, total monthly household income, smoking habits, drinking habits, exercise in daily life, a balanced diet in daily life, and mode of daily transportation.

In this stage, the provisional version of the questionnaire with five dimensions and 38 items was subjected to psychometric evaluation.

### Data collection

With the adoption of a convenient method, the relevant persons in charge of 10 comprehensive and specialized tertiary hospitals in the urban area were contacted. With the consent of the persons in charge, the link to the electronic questionnaire was forwarded to the nurses through WeChat for the issuing and collection of the electronic questionnaire.

### Statistic methods

The SPSS 22.0 software was used for statistical description and statistical inference, and two-sided test levels were adopted. A *P* value of < 0.05 was considered statistically significant.

1. The independent sample t-test or variance analysis was adopted to explore the effects of general data on the scientific fitness literacy of nurses: the independent sample t-test was used for comparisons between two groups, and the LSD method was used for post-hoc comparison in the case of homogeneity of variance, while Welch’s ANOVA test was used in the case of heterogeneity of variance. The Games-Howell test was used for post-hoc comparison between two groups.
2. Pearson correlation was used to analyze the correlation between each dimension of the influencing factors and the scientific fitness literacy of nurses.
3. Multiple linear regression was adopted to analyze the influencing factors of the scientific fitness literacy of nurses; the variables with a statistically significant difference in the univariate analysis and the factors significantly related to scientific fitness literacy were taken as independent variables. A multiple stepwise regression analysis was conducted on the scientific fitness literacy of nurses.

### Quality control

1. At the early stage of the study, a large number of literature readings were conducted; after repeated discussions with the supervisor and members of the research group, the research tools, as well as the inclusion and exclusion criteria, were determined, and pre-experiments were conducted to ensure the quality of the formal study. Before issuing the official electronic questionnaire link, the authors of this study communicated well with the person in charge of the nursing department of the hospital; the person in charge then forwarded the link to the head nurse, who distributed it among the nurses in the department that met the inclusion criteria.
2. The Questionnaire Star was used to design the electronic questionnaires; these were then distributed through the WeChat platform. Each IP address could only answer once to ensure the representativeness and authenticity of the sample. Each question was set as mandatory so that the nurses who filled in the questionnaire would not skip or miss any questions, thus ensuring that the questionnaire was completed perfectly. An anonymous form was adopted after the head nurse distributed the link to the nurses; it was required that the answers were submitted within 20 min after clicking the link.
3. After data collection, a second inspection was conducted by two members of the research group in order to eliminate unqualified questionnaires, thus ensuring validity; these included questionnaires completed in < 3 min or questionnaires with regular answers. After the elimination process, the data were entered directly.

## Results

This study began designing and compiling a questionnaire in October 2022. After expert consultation and reliability and validity testing, the final version of the questionnaire was completed in February 2023.

### Project analysis

#### Extreme value method

The total scores of the 300 questionnaires were sorted from highest to lowest. A total of 84 subjects with a total score of > 32 points were in the high-score group, and 81 subjects with a total score of < 15 points were in the low-score group. The independent sample t-test was used to test the difference in each item between the high-score and low-score groups. Except for item 6, “I am afraid of being injured during exercise, so I dare not exercise,” all items had t-values of > 3; the differences were statistically significant.

#### Item-to-total correlation method

The Pearson correlation was used to test the correlation between each item and the total score on the scale. Except for items 2 and 6, the correlation between each item of the scale and the total score was > 0.3 (Table 2).

Based on the above statistical results of the item analysis, the total correlation coefficient of item 2, “I believe that exercise can prevent the occurrence of diseases,” was < 3, and in item 6, “I am afraid of injury during exercise, so I dare not exercise,” the item analysis failed to meet the extreme value test and also failed to meet the statistical standard of the item-total score correlation coefficient test. Therefore, these two items were deleted.

### Validity test

#### Content validity

In the present study, the expert content validity assessment was adopted to test whether the content or items of the questionnaire might reflect the purpose or degree of the behavior to be measured. The results showed that the I-CVI at the total item level of the questionnaire on the influencing factors was 0.833–1.000, and the I-CVI at the scale level was 0.974, indicating good representativeness of the questionnaire.

#### Construct validity

**Fig. 2:**
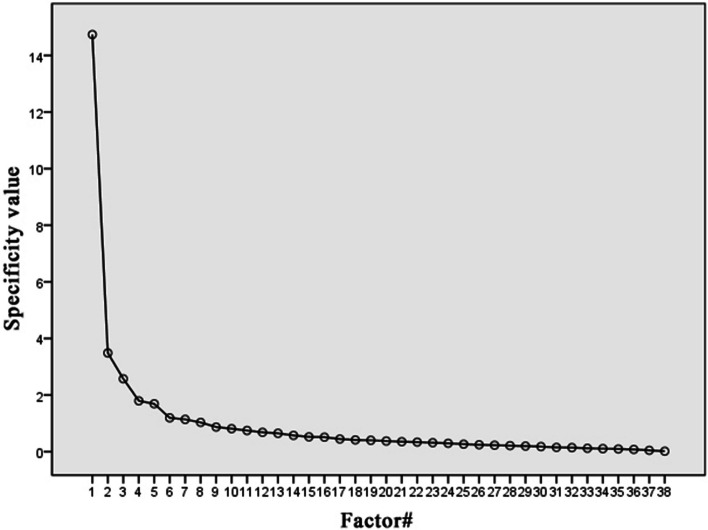
Scree plot of exploratory factors

In the present study, factor analysis was used to (1) analyze the construct validity of the influencing factors questionnaire, (2) explore the factor loadings and cumulative variance explanations of each dimension factor under different variables, and (3) further explore whether the construct validity of the questionnaire was appropriate. The results showed that the Kaiser–Meyer–Olkin value was 0.927, and Bartlett’s sphericity test had a *P* value of < 0.001; this indicated that the data were suitable for factor analysis. Based on the preconceived dimensions, the number of factors in the overall construct was set at 5, and the principal component analysis method, together with the maximum variance method, was adopted to analyze each item of the influencing factor questionnaire. The cumulative variance contribution rate was 65.84%, and the common degree of each item was > 0.3. The factor loading was > 0.4 (if the factor loading was < 4, it would not be displayed) (Table 3, Fig. 2). In terms of dimension division, the statistical results showed that the organizational dimension, policy dimension, and community environment dimension fully conformed to the preconceived dimension division; however, the statistical analysis results of the individual dimension and interpersonal dimension were different from the preconceived dimension.

**Table 3:** The analysis results of exploratory factors

	Organizational dimension	Policy dimension	Individual dimension	Community environment dimension	Interpersonal dimension	Commonality
1. I have systematically learned the knowledge and skills of scientific fitness.			0.685			0.530
3. I believe that exercise can make me look fitter.					**0.671**	0.487
4. I have a habit of exercising.			0.702			0.575
5. I like exercises.			0.500			0.485
7. A professional coach guides me on how to exercise scientifically.			0.740			0.557
8. I can schedule exercise time under any situation,.			0.751			0.595
9. I need to make myself stronger through exercise.					**0.577**	0.459
10. My family, friends, or colleagues enjoy exercises.					0.472	0.446
11. My family, friends, or colleagues often meet to exercise together.			**0.663**			0.559
12. I am able to obtain scientific fitness information through various media.			**0.430**			0.379
13. I am able to get guidance from a fitness professional before I worked out at the gym.			**0.690**			0.637
14. The community where I live often invites professionals to conduct scientific fitness education activities for residents.			**0.694**			0.618
15. Our hospital attaches great importance to the integration strategy of exercises and medicine.	0.550					0.525
16. The concepts of “exercise is a good doctor” and “exercise is good medicine” are highly recognized in our hospital.	0.679					0.537
17. Our hospital attaches great importance to the guidance of exercise rehabilitation for patients.	0.748					0.699
18. Our hospital organizes medical staff to participate in the training of scientific fitness knowledge and skills.	0.677					0.656
19. Our hospital encourages doctors to prescribe exercise rehabilitation for patients.	0.725					0.647
20. Our hospital encourages nurses to educate and guide patients in exercise rehabilitations.	0.754					0.619
21. Our hospital often organizes staff to conduct various types of fitness activities.	0.804					0.726
22. Our hospital has a place for fitness activities and is equipped with complete fitness facilities.	0.702					0.688
23. Our hospital will invite professional scientific fitness instructors to give guidance during the conduction of fitness activities.	0.687					0.705
24. Our hospital leaders are very supportive of scientific fitness exercises.	0.779					0.710
25. I am able to understand the purpose and usage of the fitness facilities in the community where I live.				0.528		0.504
26. There exists a sports ground or gym in or near my community.				0.542		0.593
27. The above sports grounds or gyms are equipped with professional fitness instructors.				0.434		0.573
28. It’s convenient for me to go from where I live to the sports ground or gym.				0.515		0.648
29. There are free gymnasiums in our community, equipped with a variety of fitness facilities.				0.845		0.848
30. The fitness and sports facilities in the community are easily accessable.				0.802		0.808
31. The environment of the sports ground or gym in the community are comfortable.				0.787		0.831
32. I learned the theory and skills of scientific fitness at all stages when I was a student.		0.572				0.652
33. I understand national or local policies and requirements for science fitness.		0.801				0.836
34. I understand the policies and requirements on exercises and medicine integration issued by the country.		0.832				0.867
35. I understand the policies and requirements of the national health management department on strengthening exercise rehabilitation for those with chronic diseases.		0.881				0.908
36. I understand the national or local environmental modification policies for scientific fitness.		0.893				0.945
37. I understand the city planning policy for scientific fitness formulated by the country or the local region.		0.904				0.958
38. I understand the policy support of the national or local medical insurance reform for the integration of physical and medical science and fitness (sports rehabilitation).		0.863				0.893

### Reliability test

A reliability test, also known as reliability analysis, can reflect the reliability and stability of the assessment tools. The most commonly used reliability coefficient, Cronbach’s α coefficient, was adopted in this study to measure the internal consistency of the questionnaire. The total Cronbach’s α coefficient of the influencing factors questionnaire was 0.955; Cronbach’s α coefficient of the individual, interpersonal, organizational, community environment, and policy dimensions were 0.782, 0.814, 0.928, 0.912, and 0.971, respectively (Table 4).

**Table 4:** Reliability test results of the questionnaire on influencing factors of scientific fitness literacy of nurses

Dimension	Item number	Cronbach’s α
personal	7	0.782
Among people	5	0.814
organization	10	0.928
Community Environment	7	0.912
policy	7	0.971
General questionnaire	36	0.955

Therefore, the official draft of the questionnaire on the influencing factors of the scientific fitness literacy of nurses with 36 items and five dimensions (individual, interpersonal, organizational, community environment, and policy dimensions) was determined. The question options were set into two categories: “yes” and “no”. In the case of selecting “no,” the subject was given 0 points. In the case of selecting “yes,” the subject was given 1 point.

### Investigation on the influencing factors of the scientific fitness literacy of nurses

#### Sampling method and sample size estimation

It was suggested in the relevant literature that the average sample size of a regional investigation required at least 500 subjects [14]. Also, in the investigation exploring the influencing factors of variables, the minimum sample size should be 5–10 times of the number of variables [14].

$$\mathrm{N }= [\mathrm{the\ number\ of\ the\ variable }\times (5-10)] \times [1+10\mathrm{\%}]$$

The present study aimed to (1) investigate the current status of the scientific fitness literacy of nurses in tertiary hospitals in Baoding and (2) explore the influencing factors. With consideration of 20% of the questionnaires being invalid, the sample size should be at least 600.

#### Questionnaire on the influencing factors of the scientific fitness literacy of nurses

Through literature review and expert consultation and based on the theoretical model of socioecology, a questionnaire on the influencing factors of the scientific fitness literacy of nurses with 36 items in five dimensions (individual, interpersonal, organizational, community environment, and policy dimensions) was designed. The question option was set into two categories: “yes” and “no”. In the case of “no,” the subject was given 0 points. In the case of “yes,” the subject was given 1 point.

#### Data collection

The convenience sampling method was adopted to select research objects. The specific sampling process was as follows: the relevant persons in charge of the municipal tertiary hospitals in the urban area were contacted, and with the consent of the persons in charge, the link to the electronic questionnaire was forwarded to the nurses in the municipal tertiary hospitals in the urban area through WeChat for the issuing and collection of the electronic questionnaire. In February 2023, a survey was conducted on the influencing factors of 2566 nurses in tertiary hospitals, and 2400 valid questionnaires were ultimately collected.

### Correlation analysis of the relationship between the individual, interpersonal, organizational, community environment, and policy dimensions and the scientific fitness literacy of nurses

The results of the pairwise Pearson correlation analysis revealed that the individual, interpersonal, organizational, community environment, and policy dimensions were all positively correlated with the scientific fitness literacy of nurses; the correlation coefficients were all > 0.3 (Table 5).

**Table 5:** Correlation analysis between different variables in each dimension and scientific fitness literacy of nurses

	Interpersonal dimension	Organizational dimension	Community environment dimension	Policy dimension	Individual dimension	The total literacy score
Interpersonal dimension	1					
Organizational dimension	0.520^**^	1				
Community environment dimension	0.584^**^	0.692^**^	1			
Policy dimension	0.555^**^	0.617^**^	0.669^**^	1		
Individual dimension	0.730^**^	0.421^**^	0.468^**^	0.448^**^	1	
The total literacy score	0.444^**^	0.372^**^	0.403^**^	0.429^**^	0.423^**^	1

^**^P < 0.01

### Multivariate analysis of the scientific fitness literacy of nurses

To further analyze the influencing factors of the scientific fitness literacy of nurses, literacy was taken as the dependent variable; furthermore, the data with general information that had statistical differences in the univariate analysis together with the five dimensions that were significantly correlated with scientific fitness literacy were taken as the independent variables. A multiple-stepwise regression equation was constructed. The results of the multiple stepwise regression equation showed that factors such as interpersonal dimension, policy dimension, balanced daily diet, individual dimension, community environment dimension, the persistence of exercise in daily life, educational background, job title, and organizational dimension, were included in the equation; the above influencing factors might explain 28.9% of the total variation of the scientific fitness literacy of nurses (the adjusted R^2^ = 0.289) (Table 6).

**Table 6:** Multiple regression analysis of scientific fitness literacy of nurses

	B	Standard coefficient(β)	*t*	*P*
Interpersonal dimension	1.544	0.116	4.1	0.000
Policy dimension	1.469	0.185	7.42	0.000
The existence of a balanced diet in daily life	5.8	0.116	6.287	0.000
Individual dimension	1.675	0.135	5.066	0.000
Community environment dimension	0.673	0.071	2.596	0.009
The persistence of exercise in daily life	3.964	0.072	3.705	0.000
Educational background	-3.197	-0.047	-2.672	0.008
Job title	-1.07	-0.042	-2.389	0.017
Organizational dimension	0.398	0.053	2.104	0.036

*F* = 109.169, *P* < 0.001, *R*^*2*^ = 0.291, Adjusted *R*^*2*^ = 0.289

### Analysis of each item in the questionnaire on the influencing factors of the scientific fitness literacy of nurses

The proportion of nurses who answered “no” in the questionnaire on the influencing factors of the scientific fitness literacy of nurses was ranked from highest to lowest to display the status quo of the existence of various influencing factors in the surveyed population (Table 7).

**Table 7:** Analysis of the influencing factors of scientific fitness literacy of nurses on each item

	Option	Frequency	% (The percentage of those with an answer of “Yes”)
With a professional coach guiding me on how to exercise scientifically	No	1823	76(24)
I can schedule exercise time under any situation,.	No	1795	74.8(25.2)
I have systematically learned the knowledge and skills of scientific fitness.	No	1769	73.7(26.3)
The community where I live often invites professionals to conduct scientific fitness education activities for residents.	No	1615	67.3(32.7)
I am able to get guidance from a fitness professional before I worked out at the gym.	No	1496	62.3(37.7)
My family, friends, or colleagues often meet to exercise together.	No	1433	59.7(40.3)
I have the habit of exercising.	No	1415	59(41)
I am able to understand the purpose and usage of the fitness facilities in the community where I live.	No	1015	42.3(57.7)
I understand the policies and requirements on exercises and medicine integration issued by the country.	No	1010	42.1(57.9)
I understand national or local policies and requirements for science fitness.	No	995	41.5(58.5)
I understand the city planning policy for scientific fitness formulated by the country or the local region.	No	992	41.3(58.7)
I understand the national or local environmental modification policies for scientific fitness	No	980	40.8(59.2)
I understand the policy support of the national or local medical insurance reform for the integration of physical and medical science and fitness (sports rehabilitation).	No	971	40.5(59.5)
I understand the policies and requirements of the national health management department on strengthening exercise rehabilitation for those with chronic diseases.	No	964	40.2(59.8)
I learned the theory and skills of scientific fitness at all stages when I was a student.	No	958	39.9(60.1)
My family, friends, or colleagues enjoy sports.	No	956	39.8(60.2)
I like sports.	No	950	39.6(60.4)
I am able to obtain scientific fitness information through various media.	No	920	38.3(61.7)
Our hospital attaches great importance to the integration strategy of exercises and medicine.	No	917	38.2(61.8)
It’s convenient for me to go from where I live to the sports ground or gym.	No	913	38(62)
The above sports ground or gym are equipped with professional fitness instructors.	No	886	36.9(63.1)
The environment of the sports ground or gym in the community are comfortable.	No	834	34.8(65.2)
Our hospital will invite professional scientific fitness instructors to give guidance during the conduction of fitness activities.	No	785	32.7(67.3)
Our hospital has a place for fitness activities and is equipped with complete fitness facilities.	No	779	32.5(67.5)
There are free gymnasiums in our community, equipped with a variety of fitness facilities.	No	761	31.7(68.3)
The fitness and sports facilities in the community are easily accessable.	No	735	30.6(69.4)
There exists sports ground or gym in or near my community.	No	730	30.4(69.6)
Our hospital organizes medical staff to participate in the training of scientific fitness knowledge and skills.	No	679	28.3(71.7)
I need to make myself stronger through exercise.	No	642	26.8(73.2)
Our hospital encourages doctors to prescribe exercise rehabilitation for patients.	No	604	25.2(74.8)
Our hospital often organizes staff to conduct various types of fitness activities.	No	515	21.5(78.5)
The concepts of “exercise is a good doctor” and “exercise is good medicine” are highly recognized in our hospital.	No	461	19.2(80.8)
Our hospital leaders are very supportive of scientific fitness exercises.	No	442	18.4(81.6)
Our hospital encourages nurses to educate and guide patients in exercise rehabilitations.	No	401	16.7(83.3)
Our hospital attaches great importance to the guidance of exercise rehabilitation for patients.	No	382	15.9(84.1)
I believe that exercise can make me look fitter.	No	185	7.7(92.3)

In order to present the existing status of various influencing factors in the survey population, the proportion of nurses who answered “no” in the questionnaire on influencing factors of scientific fitness literacy was ranked from high to low

## Discussion

### The scientific fitness literacy of nurses was influenced by multi-level and multi-dimensional factors

In the present study, the individual dimension mainly included the individual’s cognition, attitude, motivation, and self-efficacy regarding exercise; these were the internal factors of the individual. Among them, self-efficacy played a mediating role in knowledge, behavior, and skills. Choosing a favorite fitness program might help solve the problem with continuity in scientific fitness. The medical staff generally agreed with the benefits of physical exercise, and their cognitive attitudes towards the advantages of physical exercise were positive; however, there existed a lag in exercise behavior as well as a “conflict of knowledge and action.” Therefore, future work will focus on how to mobilize the internal enthusiasm of medical staff and transform it into the practice of scientific fitness and improve the self-efficacy and motivation of the scientific fitness of nurses, giving full play to their subjective initiative.

In the present study, the contribution of the interpersonal dimension to predicting the scientific fitness literacy of nurses ranked first; it was the most important influencing factor, with a significant impact on literacy. The interpersonal dimension in this survey mainly referred to social support. The more support there came from peers, friends, family members, professionals, etc., the easier it was to form the intention and motivation to exercise. Interpersonal communication on scientific fitness can be used to promote the improvement of nurses’ scientific fitness literacy. Social support provides exogenous motivation for participating in scientific fitness activities, and the more social support one obtains, the higher the selectivity and enthusiasm for fitness activities [14–16]; hence, increasing scientific fitness literacy.

The study subjects in the present study were nurses. Therefore, the organizational dimension mainly referred to the hospital, including the hospital’s emphasis on exercises and medicine integration as well as on employee fitness. The emphasis on exercise and medicine integration and the degree of support for scientific fitness by the hospital affected the provision of nurses’ exercise guidance to patients. Therefore, finding out how to improve the scientific fitness literacy of medical staff through the professional organizational structure of the hospital would be critical; it would not only help make up for the “shortcomings” of the health promotion and education of medical staff but also contribute to the improvement of national health literacy under the strategic goal of “Healthy China”. Creating a hospital fitness culture atmosphere and satisfying the needs of nurses for fitness activities would help improve the scientific fitness literacy of nurses.

The social environment significantly impacts physical exercise and is an external boost, as it includes venues and equipment. A favorable fitness environment and the degree of landscaping may facilitate positive exercise awareness and attitude as well as provide social support for physical exercise [17]. The results of the influence of this dimension on scientific fitness were consistent with the research results of Cerin et al. [18–20]. Therefore, creating a positive community fitness environment would benefit nurses’ participation in fitness exercises in the community after returning home from work, thereby improving their scientific fitness literacy.

The policy dimension is the macro system in the ecosystem and the outermost level of the socioecological model. The influence of the policy dimension was more mandatory and stable than the influence of other dimensions. The policy dimension in this study included the understanding of current fitness-related policies by the individuals and whether they were implemented in accordance with policy requirements when the nurses were students. The successful introduction of a series of policies regarding scientific fitness, with the medical and health institutions as important policy implementation units, would enable the nurse population in these institutions to continue to learn about scientific fitness-related content during the implementation of these policies, promoting the improvement of scientific fitness literacy. Therefore, hospitals should increase the publicity of scientific fitness-related policies, such as “Healthy China Action (2019–2030),” formulate practical policies, and raise awareness among nurses that they are a part of policy implementation; this should help them actively learn the contents related to scientific fitness and improve scientific fitness literacy, allowing them to provide patients with better health promotion services through exercise and play the role of scientific fitness in health promotion [21, 22].

### Other factors

In addition to the impact of the five different dimensions constructed by the socioecological model on the scientific fitness literacy of nurses, individual factors, such as gender, lifestyle, educational background, and job title, also had an impact on the scientific fitness literacy of nurses. Generally, the athletic ability, enthusiasm, and attitude toward exercise are higher in males than in females [23]. This view is consistent with our findings that male nurses may have more advantages in scientific fitness knowledge learning and application. Daily life style has an important impact on scientific fitness literacy. Maintaining a healthy lifestyle means having a higher level of health literacy, and scientific fitness is an important part of health literacy. Scientific fitness literacy is based on scientific theories and methods to guide the movement ability of the body. Studies have shown that age, gender, education level, region and exercise mode are the main factors affecting science and health literacy, and higher education level and regular exercise are closely related to better science and health literacy [24]. This explains the strong relationship between a healthy lifestyle and scientific fitness literacy. Daily life style has an important impact on scientific fitness literacy. Maintaining a healthy lifestyle means having a higher level of health literacy, and scientific fitness is an important part of health literacy. Scientific fitness literacy is based on scientific theories and methods to guide the movement ability of the body. Studies have shown that age, gender, education level, region and exercise mode are the main factors affecting science and health literacy, and higher education level and regular exercise are closely related to better science and health literacy [24]. This explains the strong relationship between a healthy lifestyle and scientific fitness literacy.

It was found in this study that the score of scientific fitness literacy was higher in nurses with a junior college degree than in nurses with an undergraduate degree and nurses with a master’s degree or above; the educational background was negatively correlated with scientific fitness literacy to a certain extent. The reason for this may be related to the nurses’ occupations. Nurses with an undergraduate or postgraduate degree need to spend energy on scientific research and teachings in addition to clinical tasks, leaving them with less time for fitness. In the analysis of influencing factors on each item, “lack of time” was an important factor affecting the scientific fitness behavior of nurses. It was also revealed in this study that the scientific fitness literacy score of nurses with the job title “nurse” was higher than in nurses with the job titles “responsible nurse” and “head nurse”; the job title was negatively correlated with scientific fitness literacy to a certain extent. This correlation may be similar to the correlation with educational background. Responsible nurses and head nurses have heavier clinical responsibilities and management tasks; they are also under relatively high pressure, making it difficult to guarantee that they will have time for fitness. As a result, the nurses with higher job titles had a low level of scientific fitness literacy.

The evaluation tools adopted in this study were all designed by the research group. Reporting bias might exist due to the lack of an examiner-rating scale or objective indicators. Additionally, the sampling subjects were drawn only from urban tertiary hospitals; therefore, the conclusions should be promoted cautiously. In the future, a large-sample multi-centered survey could be conducted to improve the representativeness of the samples, and further experimental research could be conducted according to the influencing factors of the scientific fitness literacy of nurses in order to provide a basis for the improvement of their scientific fitness literacy.

## Conclusion

The factors that affected the scientific fitness literacy of nurses involved all levels of the socioecological system (the individual, interpersonal, hospital, community environment, and policy dimensions). For nurses, the current practice of hospitals to improve the awareness of scientific fitness in nurses and provide opportunities for scientific fitness activities plays an important role in the improvement of their scientific fitness literacy. However, the lack of professional guidance, time, systematic scientific fitness knowledge and skill training, and scientific fitness culture atmosphere in the community environment hinders the improvement of the scientific fitness literacy of nurses.

In the context of the construction of a healthy China, the enthusiasm for fitness is high throughout the entire population; however, the health benefits of exercise can only be achieved by conducting scientific fitness. Based on the important role of nurses in the context of exercise and medicine integration, a systematic and comprehensive study was conducted for the first time to explore the influencing factors of the scientific fitness literacy of nurses from different levels, such as the individual, interpersonal, hospital, community, and policy dimensions, based on the socioecology theories. Improving the scientific fitness literacy of nurses would be of great significance both in their lives and in their guidance of patients.

## Data Availability

All data generated or analysed during this study are included in this article. Further enquiries can be directed to the corresponding author.
